# Volcanic sintering: Timescales of viscous densification and strength recovery

**DOI:** 10.1002/2013GL058105

**Published:** 2013-11-15

**Authors:** Jérémie Vasseur, Fabian B Wadsworth, Yan Lavallée, Kai-Uwe Hess, Donald B Dingwell

**Affiliations:** 1 Department of Earth and Environmental Sciences, Ludwig Maximilians UniversityMunich, Germany; 2 Department of Earth, Ocean and Ecological Sciences, University of LiverpoolLiverpool, UK

**Keywords:** volcanic ash, tuffisite, porosity, ultrasonic velocity, brittle, pore-emanated crack model

## Abstract

[1] Sintering and densification are ubiquitous processes influencing the emplacement of both effusive and explosive products of volcanic eruptions. Here we sinter ash-size fragments of a synthetic National Institute of Standards and Technology viscosity standard glass at temperatures at which the resultant melt has a viscosity of ∼10^8^–10^9^ Pa.s at 1bar to assess sintering dynamics under near-surface volcanic conditions. We track the strength recovery via uniaxial compressive tests. We observe that volcanic ash sintering is dominantly time dependent, temperature dependent, and grain size dependent and may thus be interpreted to be controlled by melt viscosity and surface tension. Sintering evolves from particle agglutination to viscous pore collapse and is accompanied by a reduction in connected porosity and an increase in isolated pores. Sintering and densification result in a nonlinear increase in strength. Micromechanical modeling shows that the pore-emanated crack model explains the strength of porous lava as a function of pore fraction and size.

## 1. Introduction

[2] Welding or sintering of volcanic ash and lava densification occurs by a combination of viscous flow and chemical diffusion and takes place in a variety of volcanic settings. This process is evident in rheomorphically welded ignimbrites [*Sparks et al.*, 1999], tuffisite veins [*Stasiuk et al.*, 1996; *Kolzenburg et al.*, 2012], shallow conduits [*Tuffen et al.*, 2003; *Tuffen and Dingwell*, 2005], lava flows [*Cabrera et al.*, 2011], and lava domes.

[3] High-grade (high temperature) pyroclastic density currents can also sinter during deposition, resulting in dense ignimbrites [e.g., *Smith*, 1960; *Ragan and Sheridan*, 1972; *Branney and Kokelaar*, 1992] that are sometimes mistaken for lavas in the field. In this process, a density-graded particulate flow is thought to progressively agglutinate (i.e., syn-deformational sintering) to a nonparticulate, viscously deformable flow, forming a rheomorphic ignimbrite [*Branney and Kokelaar*, 1992]. The sintering or welding intensity can be estimated from strain markers in such deposits [*Quane and Russell*, 2005, 2006]; and using existing experimental models, the timescale over which strain is accumulated can be estimated [*Russell and Quane*, 2005].

[4] Fracture and subsequent healing also occurs in shallow conduits and lava domes [*Tuffen and Dingwell*, 2005]. This phenomenon is observed at all scales and is in all likelihood integral to the structural stability of lava domes. Thermochemical, kinetic investigation of fractures in obsidian has demonstrated the efficiency of these processes [*Cabrera et al.*, 2011; *Castro et al.*, 2012]. During this process, strength can be recovered and repeated fracture and healing may take place [*Tuffen et al.*, 2003]. In some instances, fractures may be filled by fragmented particles generating tuffisite veins. Tuffisites form during brittle magmatic fragmentation, subsequent transport through, and deposition in, fracture networks. Tuffisites consist of fine-grained fragments (1μm–1mm) that relax and sinter in situ, forming diagnostic vein-filled brecciated textures [*Tuffen et al.*, 2003; *Tuffen and Dingwell*, 2005; *Kolzenburg et al.*, 2012]. *Kolzenburg et al.* [2012] have demonstrated that the strength of tuffisites can be recovered during the healing process and as such, suggested that the influence of tuffisites on the rheological, mechanical, and physical behavior of lava domes is limited to the time frame over which the vein heals.

[5] Rheological experiments have shown that during welding the apparent viscosity of the porous particulate lava progressively recovers in value to that of the liquid [*Quane and Russell*, 2003]. The rate of the process has been shown to depend on stress and melt viscosity before vitrification (at the glass transition) or crystallization [*Smith*, 1960; *Sparks et al.*, 1999; *Quane and Russell*, 2005; *Russell and Quane*, 2005; *Quane et al.*, 2009]. As the kinetics of the process is viscosity-dependent, the degree of densification could be strongly influenced by the presence of volatiles in the liquid phase [*Hess and Dingwell*, 1996] and thus by the availability of gas in the pore space to resorb into the liquid structure [*Sparks et al.*, 1999]. Rheological studies addressing welding in volcanic systems have however neglected conditions in which no external stress is applied where the fundamental mechanisms by which melt droplets coalesce yield the kinetics of the surface tension driven end-member of this process. In material sciences, these phenomena are well-studied [e.g., *Frenkel*, 1945; *Mackenzie and Shuttleworth*, 1949; *Scherer*, 1977; *Prado et al.*, 2001]. Here we build on previous rheological studies to investigate the kinetics of sintering and magma densification to constrain in turn strength recovery during healing. Micromechanical analysis is then employed to provide a failure criterion for porous lavas.

## 2. Experimental Methods

[6]The process of glass particle sintering has been investigated using the standard reference material SRM 717a, which is a borosilicate glass from the National Institute of Standards and Technology (NIST, USA). This material has been selected as it has a well-constrained viscosity-temperature dependence, a precise description of the calorimetric glass transition interval (506–520°C at 10°C min^−1^ natural cooling rate), a determined fracture toughness (0.7MPa m^1/2^; *Wiederhorn* [1969]), and it has been used extensively in background studies to constrain volcanic processes [*Hess et al.*, 2007; *Robert et al.*, 2008; *Whittington et al.*, 2009; *Cordonnier et al.*, 2012]. This material does not crystallize, degas, or show liquid-liquid immiscibility at the experimental conditions and timescales.

**Figure 1:**
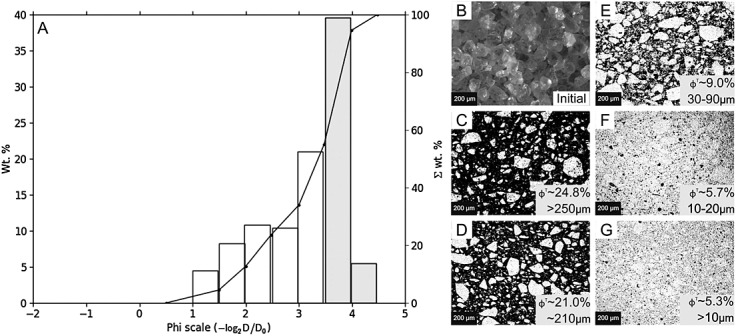
Experimental material used in this study. (a) Grain size distribution for the starting material (63≤*φ*≤355μm). (b) Photomicrograph (reflected light) of the starting material of unconsolidated sieved 100≤*φ*≤150μm grain size powder of NIST 717a standard glass. (c–g) Binary false-color thin section photomicrographs of samples sintered at 650°C for incremental times. Black represents pores and white the glass.

[7]We crushed the glass to a powder using a concussion mill for intervals of 10–15s and measured the resultant grain size distribution by sieving with *φ*/2 intervals (Figure 1). We systematically filled alumina ceramic crucibles (44mm in diameter and 75mm in height) with the unsieved powder, ensuring maximum packing by gently tapping the powder flat every time we poured a few millimeters of powder in the crucible. The maximum packing is a function of the grain size distribution, sorting, and clast angularity for heterogeneous powder populations [*Evans and Gibson*, 1986]. The sample-laden crucibles were heated at 10°C min^−1^ to isotherms above the glass transition and at which the melt viscosity is 10^8.04^ and 10^9.35^ Pa s, respectively. Sintering took place during dwells of 0.5 to 10h. After sintering, the samples were cooled at a slower rate of 5°C min^−1^ to minimize cracks induced by the thermal contraction of the sample. Note that the sintering times shown here are the dwell time at the isotherm and do not include the heating and cooling portions of the sample excursions to and from high temperature. Due to the fast heating rate sintering occurring during the heating portion above the glass transition, temperature is considered negligible (“supporting information Figure S1”). The samples show no preferential compaction at the base nor cracking both of which would affect the bulk porosity. The densified products were drilled from the crucible to sample cores of 25 × 50mm for further physical and mechanical analysis.

[8] A physical description of the welded products requires an accurate description of the porous network. The connected porosity of the samples was measured by helium pycnometry using the Micromeritics Accupyc 1330. The total porosity *φ*^*T*^ was calculated from the density of the bulk sample *ρ*_bulk_ and the known density of the standard glass *ρ*_*g*_ using 
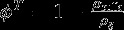
 where *ρ*_bulk_ is dependent on the mass and volume of the sample. The isolated porosity was estimated from the difference between the total and connected porosities. The density of glass is dependent on the cooling rate at which the melt crossed the glass transition, and therefore, we use a corrected glass density for the post-experimental samples given the cooling rate of ∼5°C min^−1^.

[9] Ultrasonic wave velocity was measured using a pulse generator (Agilent Technologies 33210A, 10MHz function/waveform generator) connected to piezoelectric transducers (with a resonant frequency of <1MHz) and an oscilloscope (Agilent Technologies DSO5012A). The onset of *P* wave arrival at the receiver was individually picked as the first deviation from the baseline signal.

[10] Oriented thin sections were obtained in the axial plane of the sintered samples, and photomicrographs were recorded using an optical microscope in plane-polarized light (Figure 1). The images were converted to binary, allowing for automatic thresholding of hues and gray scales to black and white. The minimum and maximum pore sizes were measured. These measurements were of connected vesicle widths in the case of the poorly sintered samples and of isolated vesicle radii in the case of the well-sintered samples.

[11] The uniaxial compressive strength of the prepared porous magmatic suspensions was determined in a high-load, high-temperature uniaxial press (see *Hess et al.* [2007] for details of the apparatus). The strength tests were performed on a relaxed (liquid) magma at a temperature of ∼535°C (i.e., above the calorimetric glass transition interval). At this temperature, a negligible amount of viscous sintering occurred on the timescale of the experiment due to the relatively high viscosity of the suspension (in contrast to the sintering experiment conditions). We loaded the porous magma up to failure at a constant strain rate of ∼10^−3^*s*^−1^ to ensure deformation in a purely brittle regime. In each experiment, the component of strain, which could not be viscously relaxed, resulted in a stress accumulation that triggered brittle failure; the peak axial stress was recorded as a measure of compressive strength.

## 3. Results: Densification and Healing of Ash by Sintering

**Figure 2:**
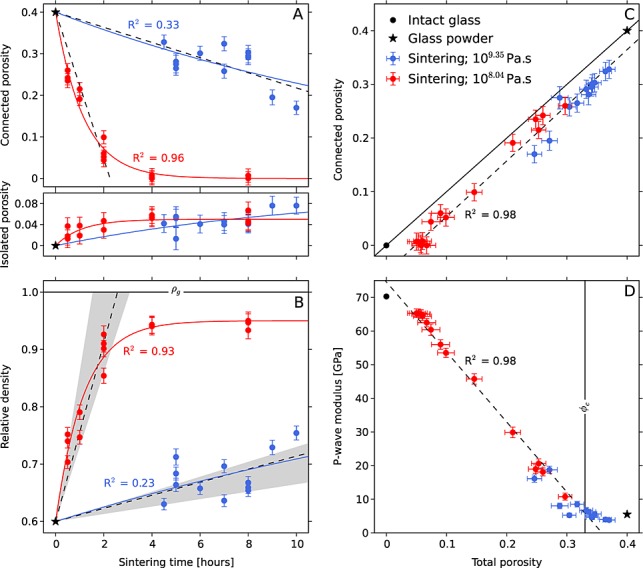
Results for porosity and density evolution in the sintered sample suite. (a) The evolution of connected (top) and isolated (bottom) porosity with sintering time with best-fit curve to models (see text). (b) Results for the evolution of relative density with sintering time. (c) The relationship between total and open porosity. (d) The relationship between the *P* wave modulus 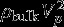 and the total porosity. Note the critical porosity *φ*_*c*_ [*Nur et al.*, 1998] occurs at the deviation from the linear trend at high porosities (∼33*%* total porosity).

[12] The total and connected porosities decrease during sintering whereas isolated porosity increases (Figure 2a). The rate of porosity change is influenced by the sintering temperature. At 650°C, the liquid has a viscosity of 10^8.04^ Pa s and the connected porosity decreases from the starting maximum packing value of ∼0.4 to 0 within 4 h. Simultaneously, the isolated porosity increases from 0 to 0.05. At 600°C, the melt viscosity is 10^9.35^ Pa s and over the experimental time of 10h, the connected and isolated porosities evolve from ∼0.4 to 0.2 and 0 to 0.07, respectively (Figure 2a). Relative density—the standard metric of sintering in ceramics and glass-technology studies—is inversely proportional to the total porosity, and we observe that the preservation and accumulation of isolated porosity prevent the recovery of the defect-free glass density (Figure 2b). The relationship between the connected porosity and the total porosity is subparallel to the isoline (total porosity = connected porosity), whereas the vertical deviation from the isoline is given by the isolated porosity (Figure 2c).

[13] The connected porosity is linearly proportional to the inverse of the measured ultrasonic *P* wave velocity (Figure 2d). The *P* wave modulus is given by 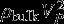, where *ρ*_bulk_ is the bulk sample density and *V*_*P*_ is the *P* wave velocity. Therefore, the *P* wave modulus is also linearly inversely proportional to porosity until the granular threshold value [*Nur et al.*, 1998]. This threshold is constrained here to a porosity of 0.33 (Figure 2d).

**Figure 3:**
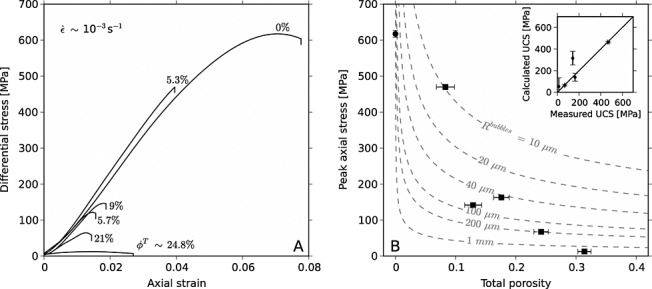
Strength and micromechanics during sintering. (a) The axial stress and strain resulting from an experimental strain rate of 10^−3^s^−1^. (b) Uniaxial peak stress at failure (proxy for the uniaxial compressive strength) during constant strain rate experiments. Displayed are the predicted isopore lines for different radii from which cracks initiate in the pore-emanating crack model [*Sammis and Ashby*, 1986; *Zhu et al.*, 2011]. The correlation between the peak failure stress measured and the uniaxial compressive strength predicted by the pore-emanating crack model for pore sizes measured in similarly sintered samples (b inset) (see Figure 1).

[14] High temperature uniaxial compressive strength tests show that stress accumulation is mostly elastic, and that failure is preceded by a minor amount of strain hardening, highlighting the predominantly brittle nature of lava at such viscosity and high-strain rate (Figure 3a). The uniaxial compressive strength (measured at 10^−3^ s^−1^) increases with sintering time and thus decreases nonlinearly with total porosity (Figure 3b).

[15] Microstructural analysis reveals further details of the sintering process. We note a rapid coarsening of the ash fragments due to agglutination. This densification process results in turn in an overall decrease in average pore size, which evolves from ∼1 to ∼0.01mm over the sintering timescale (Figure 1). The findings are consistent with the general observation that sintering results in viscous pore collapse, densification, and strengthening of porous lava.

## 4. Interpretation of Sintering Timescales and Mechanisms

[16] We interpret our data with a scenario in which viscous sintering from a granular to a dense, pore-free material is a three-stage process for Newtonian fluids (Figure 2). First, following the transition from a glass to a liquid, viscous relaxation of the ash fragments induces necking at their interfaces, forming an agglutinated frame. Second, this agglutinated frame yields an interconnected porous network, which is inherently unstable with respect to surface tension in the absence of pore pressure. This framework viscously collapses causing the majority of the volumetric strain via porosity reduction. Third, the closure of the porous network yields isolated pores (i.e., gas bubbles) suspended in the liquid phase, which act to reduce the density from that of the pure liquid (Figure S2).

[17] These stages have been approximated by theoretical and empirical relationships. Neck formation is well described by the Frenkel and Scherer sintering models, and variations of these studies which states that relative density will increase with time between relative densities of 0.3 and 0.9 [*Frenkel*, 1945; *Scherer*, 1977]. We find that a combination of their approaches yields an empirical, linear relationship valid for the range of relative densities over which neck formation dominates 0.3–0.9. This model, which neglect stress, requires that viscous sintering is characterized by a dominant sintering timescale *τ*_*s*_. *Uhlmann et al.* [1975] suggest that this timescale is related to the melt viscosity *η*, the melt-vapor interfacial tension *γ*, and the initial radius of the sintering particles 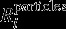



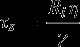
(1)

[18] Values of *τ*_*s*_ correspond to the timescale over which the dominant process acts. The driving stress for deformation is derived from the surface tension and the interconnected pore geometries. The fact that we can approximate the evolution of porosity and density using equation (1) implies that the deformation is viscous and that diffusive neck growth is not the dominant transport mechanism. Equation (1) is identical to the approximation for the viscous relaxation time of a bubble in a melt defined by *Oldroyd* [1953], in which 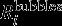 is the initial bubble radius. We note that there is a packing-dependent proportionality between *R*_*i*_ of statically sintering particles in a granular material and 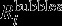 of bubbles in the resultant viscously relaxing suspension. This consideration permits us to describe a continuum in the processes of sintering and bubble relaxation as the material progresses from granular to a suspension medium. As such, the data can be empirically approximated by the exponential expression


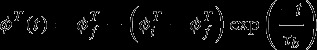
(2)

where 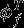 is the final total porosity and *τ*_*b*_ is the timescale of densification which is dominantly related to the bubble collapse and relaxation timescale. Because the total porosity includes isolated and connected pores, and because the results for viscous sintering at 650°C show that the connected porosity decays to zero (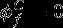; Figure 1a), equation (2) becomes 
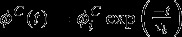
.

[19] Injecting equation (2) into the total porosity calculation based on density, one can provide the relative density evolution with time


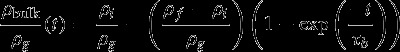
(3)

[20] We use a least squares regression to fit for values of *τ*_*s*_ and *τ*_*b*_ for each experimental temperature using equation (S1) and equation (3), respectively (Figure 2b). This operation yields a best-fit controlling grain size and bubble size for each stage of sintering. We note that *R*^particles^/*R*^bubbles^ at both experimental temperatures is ∼2–3 (Table S1), which is consistent with the pore space radii expected between grains of a heterogeneous population at maximum packing.

[21] For a known viscosity of 10^9.35^ and 10^8.04^ Pa s at 600 and 650°C, respectively, and a melt surface tension of 0.3N m^−1^ for borosilicate e-glass of a similar composition [*Kraxner et al.*, 2009], we find that the best-fit timescale of sintering for our data relates to a dominant particle radius of 25.7–37.6 μm. This particle size range is consistent for both experimental temperatures and is in very good agreement with the grain size analysis, which indicates that the most frequent particle sizes are in the range of 31.5–45.0 μm and the finest fraction is <31.5μm (grey shaded region in Figure 1a). We therefore suggest that for unimodal distributions, it is the finest grain sizes that dominate the timescale of effective sintering because those grains will occupy the interstices of larger grains and share the most contact surface area for viscous neck formation. *Prado et al.* [2001] also concluded that equation (1) holds as the finest particles cluster in pore spaces between larger particles and control sintering rates due to the high stress at their surface driving sintering.

[22] The ultrasonic *P* wave velocities are linearly dependent on total porosity, below a critical porosity threshold (here ∼0.3), that is, the porosity at which the solid phase of the material is no longer load bearing and the bulk material behaves in a granular manner at low loads [*Nur et al.*, 1998]. This relationship is well established and suggests that the progressive sintering of fragments consistently densifies the material to below the theoretical critical porosity (Figure 2d). The porosity range of the initial starting material is above the threshold, as it is granular.

## 5. Strength Recovery by Sintering and Densification

[23] Sintering and densification were observed to result in strength recovery (Figure 3a); with sintering time approaching *τ*_*s*_, the strength of single-phase, defect-free melt (glass) was recovered. As the porous structure of our medium mainly consists of pores (instead of cracks), we tested the applicability of the pore-emanated crack model to constrain compressive strength of porous lava [*Sammis and Ashby*, 1986]. *Zhu et al.* [2011] provide an analytical approximation for the pore-emanated crack model as follows:


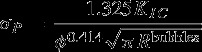
(4)

where *σ*_*P*_ is a compressive strength (MPa) and *K*_*IC*_ is the fracture toughness or the stress intensity factor (MPa m^1/2^) of glass through which a fracture needs to propagate to achieve complete failure. Note that fractures propagate by the conversion of strain energy to fracture surface energy; here, viscous dissipation of strain energy above the calorimetric glass transition is negligible, as the viscosity and strain rate are high: relaxation would require >100s but the strength test is performed in ca. 5s. This ensures deformation in a purely brittle regime. Combining measured porosities and estimated values of *K*_*IC*_ for intact, defect-free borosilicate glass of a similar composition to NIST SRM 717a of ∼0.7MPa m^1/2^ [*Wiederhorn*, 1969], we can approximate the relationship between the pore radius *R*^bubbles^ and compressive strength, using equation (4) (see grey dashed lines in Figure 3b). This modeled relationship agrees with our experimental data (Figure 3b inset) as poorly sintered samples with a high fraction of relatively large (∼1mm) pores, have a compressive strength appropriate to their pore size; similarly, highly-sintered samples with a low fraction of relatively small (∼10μm) pores have a compressive strength comparable to the modeled strength. This comparative analysis suggests that the pore-emanated crack model is wholly applicable to the strength of bubbly magma as a function of pore fraction and size at the porosities investigated here.

## 6. Discussion

[24] The results presented in this study show that surface tension is capable of sintering ash particles and densifying porous lavas. Limitations in our experiments remain however as, in nature, sintering at low pressures in conduits may be accelerated if volatiles are resorbed into the melt, locally lowering the viscosity [*Hess and Dingwell*, 1996] and facilitating neck formation. However, sintering in surficial deposits, such as ignimbrites, may occur in the presence of air, which has a low solubility in silicate melts and is therefore not a significant viscosity-forcing factor (Castro, personal communication, 2013). Additionally stress, whether compressive or tensional, can contribute to the total stress forcing densification. The results nonetheless demonstrate the near-static limiting conditions under which these processes can operate and may help provide a basic constraint on condition of sintering and densification of eruptive products in nature.

### 6.1 Plug Densification and Stiffening

[25] Sintering and densification may be important processes occurring during lava dome extrusion. The porosity of lava domes varies widely (0–80%) [*Castro and Cashman*, 1999], and the lava commonly undergoes cycles of fracture and subsequent healing with or without the presence of tuffisite ash fragments [*Tuffen and Dingwell*, 2005; *Kolzenburg et al.*, 2012]. During magma fragmentation, the fragments, which are not ejected from the shallow conduit, will sinter, heal, and recover strength. Although our experiments only considered uniaxial compressive stress (neglecting confining stress which accelerates compaction and pore pressurization but counteracts dilatation), application of the aforementioned sintering timescale relationship suggests that healing is indeed very rapid (e.g., minutes to hours) for crystal-free melts, as speculated by *Kolzenburg et al.* [2012], who were dealing with crystal-rich tuffisites which relax at a presumably lower rate. We note that even at surficial stress conditions a significant strength recovery can be achieved within the initial 15% densification. Magmastatic stress and differential stress from magma ascent, which are greater than the stress imparted by surface tension, will accelerate this process. Thus, the strength of a lava dome plugging a conduit should be seen as transient and requires knowledge of the porous network in real time if we wish to accurately constrain the eruption style [e.g., *Edmonds and Herd*, 2007].

### 6.2 Volcanic Ash Sintering in Rheomorphic Flows

[26] The description of sintering in natural volcanic settings is typically referred to as welding and only occasionally is the term “sintering” used to describe the low-grade end-member of a welding continuum [*Wilson and Hildreth*, 2003]. *Grunder and Russell* [2005] suggest that in fact welding only applies to sintering that is coupled with flattening, compaction, or stretching of pyroclasts. In the ceramics and glass science literature, the description of sintering refers to the entire continuum and encompasses the diffusive and viscous components depending on the material state.

[27] Most volcanic ash is dominantly composed of glass and so any scenario where volcanic ash is deposited close to or above the glass transition interval or where ash is subjected to a trajectory of reheating above the glass transition interval will result in a degree of viscous sintering dependent on the ratio of melt drop radius and surface tension (e.g., equation (1)). Pervasive ductile deformation structures in so-called welded ignimbrites deposited from pyroclastic density currents are interpreted to result from the continued shear stress imposed by flow of the overlying mass on viscously deformable lava-like flows [*Branney and Kokelaar*, 1992; *Manley*, 1995]. The bedload suspended in the pyroclastic density currents, which sinter upon deposition, is estimated to range between very fine ash (μm) and blocks (cm–m). Therefore, understanding of the grain size influence is critical for correctly estimating sintering timescales or temperatures of emplacement. The normalized density of such deposits has been used to rank the strain associated with sintering and compaction [*Smith*, 1960; *Wilson and Hildreth*, 2003; *Quane and Russell*, 2005]. Normalized densities for nonwelded material ranges from 0.3 to 0.4, whereas foliated and welded vitrophyric material ranges from ∼0.7 to 1.0 (welding intensity rank I–VI) [*Quane and Russell*, 2005]. Application of our modeled relationship provides a lower constraint of the timescale of the sintering interval over which porosity can be reduced to the values observed in the deposits under ambient pressure conditions, that is, neglecting the load of the overlying flowing mass. These data suggest that for a pyroclastic density current containing suspended fragments of super-cooled silicate liquid with long relaxation times, the sintering time upon deposition can be approximated by equation (1). Some authors invoke a geometrical parameter to approximate the sintering fragments to spheres, which in cases of low-angularity particles may be more appropriate [e.g., *Scherer*, 1977]. Thus, our considerations complement previous studies and provide detailed insights into the relationship between the grain size and the melt properties which should be incorporated into the rheological relationships developed for volcanic welding [e.g., *Quane et al.*, 2009].

[28] If we consider a pyroclastic deposit in its entirety, an overburden stress of 10^5^ Pa is predicted for a deposit ∼10m, which exceeds surface tension and thus induces compressive flattening and foliation during sintering [*Russell and Quane*, 2005]. However, densely welded ignimbrites commonly result from progressive aggradation from sustained density currents [*Branney and Kokelaar*, 1992] and therefore the overburden is an end-state and sintering, initiated at low axial stress and would be partially controlled by the processes we describe.

## 7. Concluding Remarks

[29] Rheological experiments have demonstrated that viscous sintering of volcanic ash at ambient pressure conditions is dominantly controlled by melt viscosity, interfacial tension between pores and melt, and grain size. Viscous sintering from granular material to a homogenous melt is a continuum process involving the evolution from sintering to melt pore collapse. We show that during viscous sintering to a texturally homogenous liquid, the resultant liquid progressively recovers strength. Micromechanical modeling concludes that the strength of bubbly magma can be estimated from the pore-emanated crack model.
