# Supplementary material for: Volcanic sintering: Timescales of viscous densification and strength recovery
Source: Geophys Res Lett. 2013 Nov 15;40(21):5658–64. doi: 10.1002/2013GL058105 (PMC4373153; doi:10.1002/2013GL058105)
Supplement: Supplementary file 3 [file grl0040-5658-sd3.pdf]

Auxiliary material for

Volcanic sintering: Timescales of viscous densification and strength recovery

Jérémie Vasseur,[1] Fabian B. Wadsworth,[1] Yan Lavallée,[2]

Kai-Uwe Hess,[1] and Donald B. Dingwell[1]

[1]Earth & Environmental Sciences, Ludwig-Maximilians-University, Munich, Germany.

[2]Earth, Ocean & Ecological Sciences, University of Liverpool, Liverpool, U.K.

Geophysical Research Letters, 2013

Empirical linear relationships

During the first stage of the sintering process (0.3–0.9 relative density), neck formation dominates and we derive an empirical, linear relationship, such that:

$$\frac{\rho_{bulk}}{\rho_g}(t) = \frac{\rho_i}{\rho_g}\left(1 + \frac{t}{\tau_s}\right) \tag{1}$$

where ρ_i is the initial bulk sample density, t is time since the onset of the isotherm and τ_s is the characteristic timescale of sintering. Injecting Eq. 1 into the total porosity calculation based on density, one can derive the total porosity as a function of time:

$$\phi^T(t) = \phi_i^T\left(1 - \frac{t}{\tau_s}\right) \tag{2}$$

where ϕ_i^T is the total porosity at $t = 0$. Eq. 1 and Eq. 2 show that the initial stage of isothermal viscous sintering can be approximated by a linear relationship with time.

Table 1. Melt parameters known or estimated for use in sintering models (see text) and model outputs.

Experimental temperature °C	Melt viscosity Pa.s	Melt surface tension N.m^{-1}	τ_s hours	R_i^s μm	τ_b hours	R_i^b μm	R_i^s/R_i^b
600	$10^{9.35}$	0.3	52.7	25.7	26.8	13.1	∼2
650	$10^{8.04}$	0.3	3.9	37.6	1.2	11.4	∼3.3

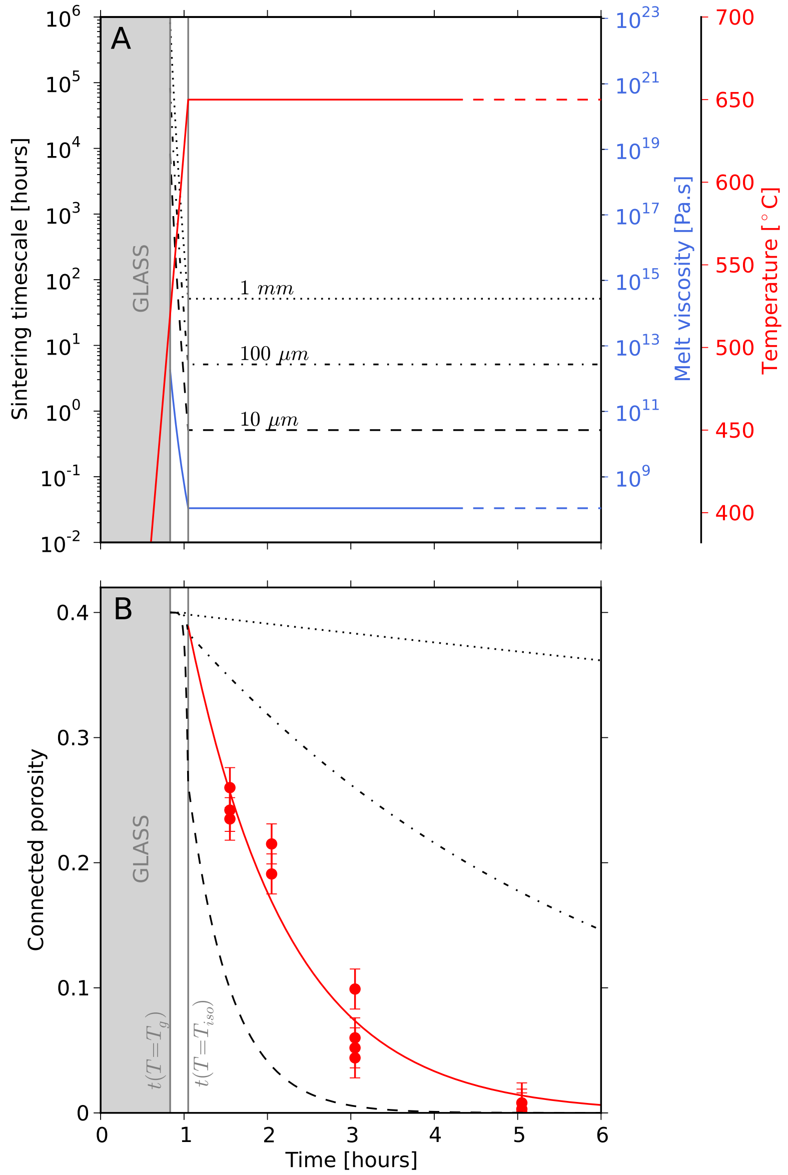

Figure 1. Model results for an experiment conducted at 650 °C. (A) The evolution of temperature, melt viscosity and modeled sintering timescale τ_s for three grain sizes as a function of time. Note the non-linear decrease in melt viscosity during heating and, consequently, the effect on the sintering timescales. (B) The evolution of connected porosity for the aforementioned three grain sizes and the best fit model (red line) to our sintering results (red circles). Note that the porosity is not predicted to significantly reduce during the heating segment of the experiment at the experimental heating rate of $10\,^{\circ}$C.min^{-1}.

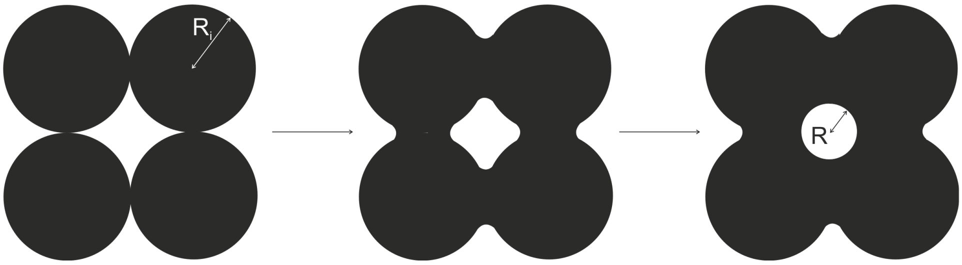

Figure 2. Schematic cartoon of the simplified spherical case of the sintering process. Sintering is a 3-stage process where (1) supercooled silicate melt droplets form necks at grain-grain contacts viscously; (2) the necks widen and encroach on the interconnected pore network; and (3) the porous network is closed leaving suspended isolated pores that relax to spherical.
